# Comparisons of developmental processes of air-breathing organs among terrestrial isopods (Crustacea, Oniscidea): implications for their evolutionary origins

**DOI:** 10.1186/s13227-024-00229-z

**Published:** 2024-07-18

**Authors:** Naoto Inui, Toru Miura

**Affiliations:** Misaki Marine Biological Station, School of Science, The University of Tokyo, Misaki, Miura, Kanagawa 238-0225 Japan

**Keywords:** Appendage, Dorsal respiratory fields, Hemolymph, Homology, Pleopodal lung, Terrestrialization, Uncovered lungs

## Abstract

**Background:**

The acquisition of air-breathing organs is one of the key innovations for terrestrialization in animals. Terrestrial isopods, a crustacean lineage, can be interesting models to study the evolution of respiratory organs, as they exhibit varieties of air-breathing structures according to their habitats. However, the evolutionary processes and origins of these structures are unclear, due to the lack of information about their developmental processes. To understand the developmental mechanisms, we compared the developmental processes forming different respiratory structures in three isopod species, i.e., ‘uncovered lungs’ in *Nagurus okinawaensis* (Trachelipodidae), ‘dorsal respiratory fields’ in *Alloniscus balssi* (Alloniscidae), and pleopods without respiratory structures in *Armadilloniscus* cf. *ellipticus* (Detonidae).

**Results:**

In *N. okinawaensis* with uncovered lungs, epithelium and cuticle around the proximal hemolymph sinus developed into respiratory structures at post-manca juvenile stages. On the other hand, in *Al. balssi* with dorsal respiratory fields, the region for the future respiratory structure was already present at manca 1 stage, immediately after hatching, where the lateral protrusion of ventral epithelium occurred, forming the respiratory structure. Furthermore, on pleopods in *Ar.* cf. *ellipticus*, only thickened dorsal cuticle and the proximal hemolymph sinus developed during postembryonic development without special morphogenesis.

**Conclusions:**

This study shows that the respiratory structures in terrestrial isopods develop primarily by postembryonic epithelial modifications, but the epithelial positions developing into respiratory structures differ between uncovered lungs and dorsal respiratory fields. This suggests that these two types of respiratory structures do not result from simple differences in the degree of development. Future analysis of molecular developmental mechanisms will help determine whether these are the result of heterotopic changes or have different evolutionary origins. Overall, this study provides fundamental information for evolutionary developmental studies of isopod respiratory organs.

## Background

Terrestrialization is one of the key innovations in the evolutionary history of animals. Terrestrial species are known to have acquired several novel traits that are not found in ancestral aquatic species, such as hard skeletal systems that resist gravity, protective shells or skins that protect against dryness and ultraviolet light, and reproductive systems that do not require water [1, 2]. One of the most important changes is in the respiratory system [3]. Major animal phyla colonizing land, such as vertebrates, arthropods and molluscs, independently acquired air-breathing organs [3–7]. However, in many cases, the detailed evolutionary processes of these traits remain unclear because of the extinction of species that had the ancestral or transitional traits [8–10]. Especially in arthropods, which are the most successful land colonizers, many lineages lack aquatic or semiterrestrial species with ancestral traits [6, 10].

To study the evolution of respiratory organs, terrestrial isopods (Crustacea, Isopoda, Oniscidea) are interesting examples because they have evolved a diversity of breathing organ during terrestrialization. Terrestrial isopods are a unique group in that they have extant species that represent each evolutionary stage of the water-to-land transitions [11]. Isopod species inhabiting arid terrestrial environments have acquired novel respiratory structures called the pseudotrachea/lung inside their abdominal appendages, pleopods, for air breathing [11, 12].

The morphology of respiratory structures in isopods are diversified in the most-derived terrestrial isopod lineage, i.e., Crinocheta. The isopod respiratory structures can be classified into three types: dorsal respiratory fields, uncovered lungs, and covered lungs [12, 13]. Simple dorsal respiratory fields are found in species of relatively basal lineages inhabiting wet environments, while species with complex covered lungs are known in derived lineages inhabiting arid terrestrial environments [13, 14]. Therefore, it was suggested that the three types of isopod respiratory structures probably share the same origins, which evolved to dorsal respiratory fields, and then to uncovered lungs, and finally to complex, desiccation-resistant covered lungs [15]. However, the same type of respiratory organs is seen in several different isopod families [16, 17], so that the evolutionary processes and the homology between organs in different isopods have remained controversial [14, 15, 17, 18].

Understanding developmental mechanisms is crucial for studying morphological evolution [19]. However, despite the importance and diversity of the morphologies of respiratory structures in terrestrial isopods, few developmental studies of these structures have been conducted [20]. Although postembryonic development of covered lungs in *Porcellio scaber* was described [18, 20], there are only limited descriptions of uncovered lung development with respect to external morphology [15, 21], and nothing is known about development of dorsal respiratory fields.

**Fig. 1:**
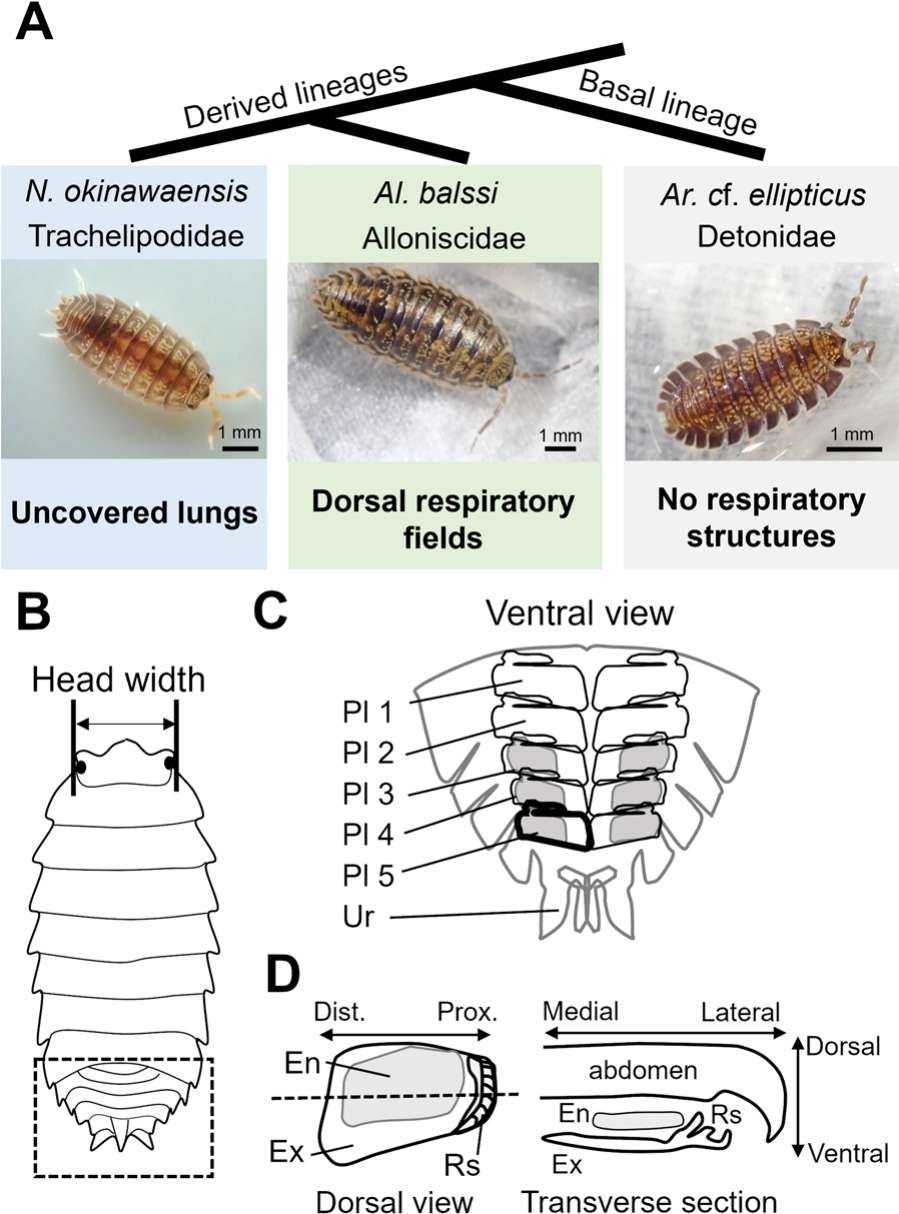
Material isopod crustaceans (**A**) and schematic diagrams of their respiratory organs (**B–D**). **A** *Nagurus okinawaensis* with uncovered lungs, *Alloniscus balssi* with dorsal respiratory fields and *Armadilloniscus* cf. *ellipticus* without respiratory structures were used in this study. Phylogenetic relationships were based on previous studies [23, 25]. **B** Dorsal view of isopods. Dashed square indicates the position of abdomen (**C**). Head width was measured as the indicator of body size. **C** Ventral view of a female abdomen. The first and second pleopods lack endopods in female. Bolded area indicates one pleopod (**D**). **D** Pleopod structures. The respiratory structures are located on the proximal (lateral) side of exopods of pleopods. Dashed line indicates the location of a transverse plane. *En* endopod, *Ex* exopod, *Dist* distal, *Prox* proximal, *Pl* pleopod; *Ur* Uropod

In this study, therefore, to gain insights into the evolutionary trajectories of these respiratory structures, we performed histomorphological studies on the development of these latter two types of respiratory structures. Uncovered lungs in *Nagurus okinawaensis* Nunomura, 1992 (Trachelipodidae) and dorsal respiratory fields in *Alloniscus balssi* (Verhoeff, 1928) (Alloniscidae) were observed during postembryonic development (Fig. 1). In addition, to assess possible ancestral states, the pleopods of *Armadilloniscus* cf. *ellipticus* (Harger, 1878), which belongs to the basal family Detonidae in Crinocheta, were also observed (Fig. 1). Based on these observations, together with previous findings, the evolutionary relationships of these respiratory organs are discussed.

## Results

### Uncovered lungs in *Nagurus okinawaensis*

#### Adult structure

**Fig. 2:**
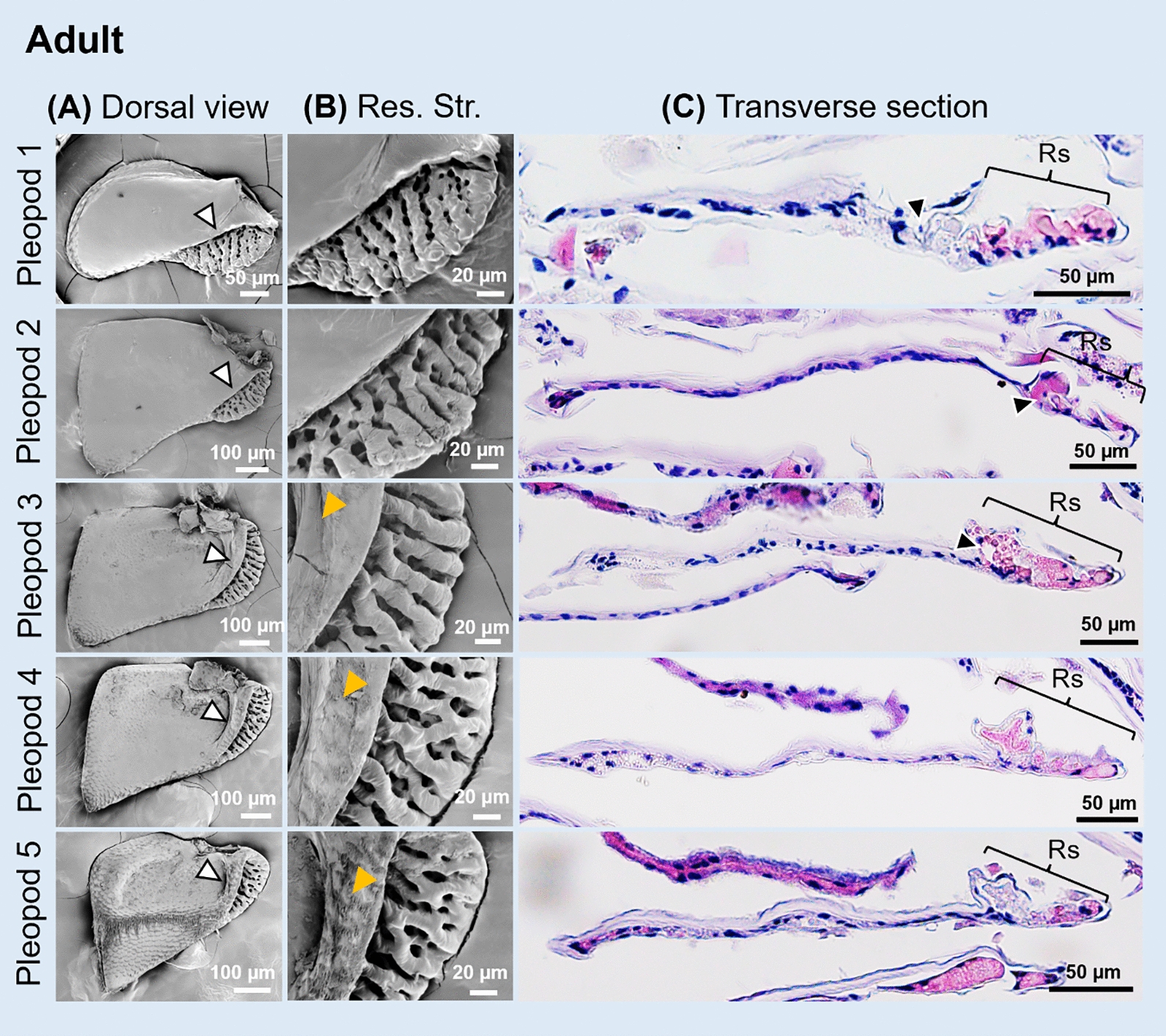
Adult pleopods of *N. okinawaensis.* **A** Dorsal view of exopods of pleopods in SEM images. White arrowheads indicate respiratory structures. **B** Enlarged views of respiratory structures (uncovered lungs). Orange arrowheads indicate swollen ridges. **C** Transverse sections of the pleopods. Respiratory structures located in proximal sides. *Res*. *Str*. and *Rs*, respiratory structure

Scanning electron microscopy (SEM) showed that, in *N. okinawaensis,* adults had highly wrinkled respiratory structures on exopods of all the pleopods (Fig. 2A–B, white arrowheads). All of the respiratory structures were located on the proximal lateral side of each pleopod. On the third to fifth pleopods, these structures were adjacent to swollen ridges (Fig. 2B, yellow arrowheads).

In addition, histological observations on transverse sections of the pleopods were carried out. The dorsal side of the exopods was covered with thicker cuticle compared to the ventral side, but the respiratory structures closer to the dorsal sides were covered with a finely furrowed epithelium and thin cuticle (Fig. 2C). Some of these furrows in the structures were deeply invaginated and formed tubular structures (Fig. 2C, black arrowheads). Inside these structures, hemolymph sinuses were observed and sinuses were partially stained with eosin (Fig. 2C). In the first and second pleopods, some parts of the respiratory structures were covered by the dorsal epithelium, whereas in the third to fifth pleopods they were not covered and were continuous with swollen ridges (Fig. 2C). The location of the hemolymph sinuses corresponded to the ridges (Fig. 2B).

#### Developmental process

The morphological structures of the first, second and third pleopods were observed during postembryonic development, from the first manca stage to the post-manca juvenile stages.

**Fig. 3:**
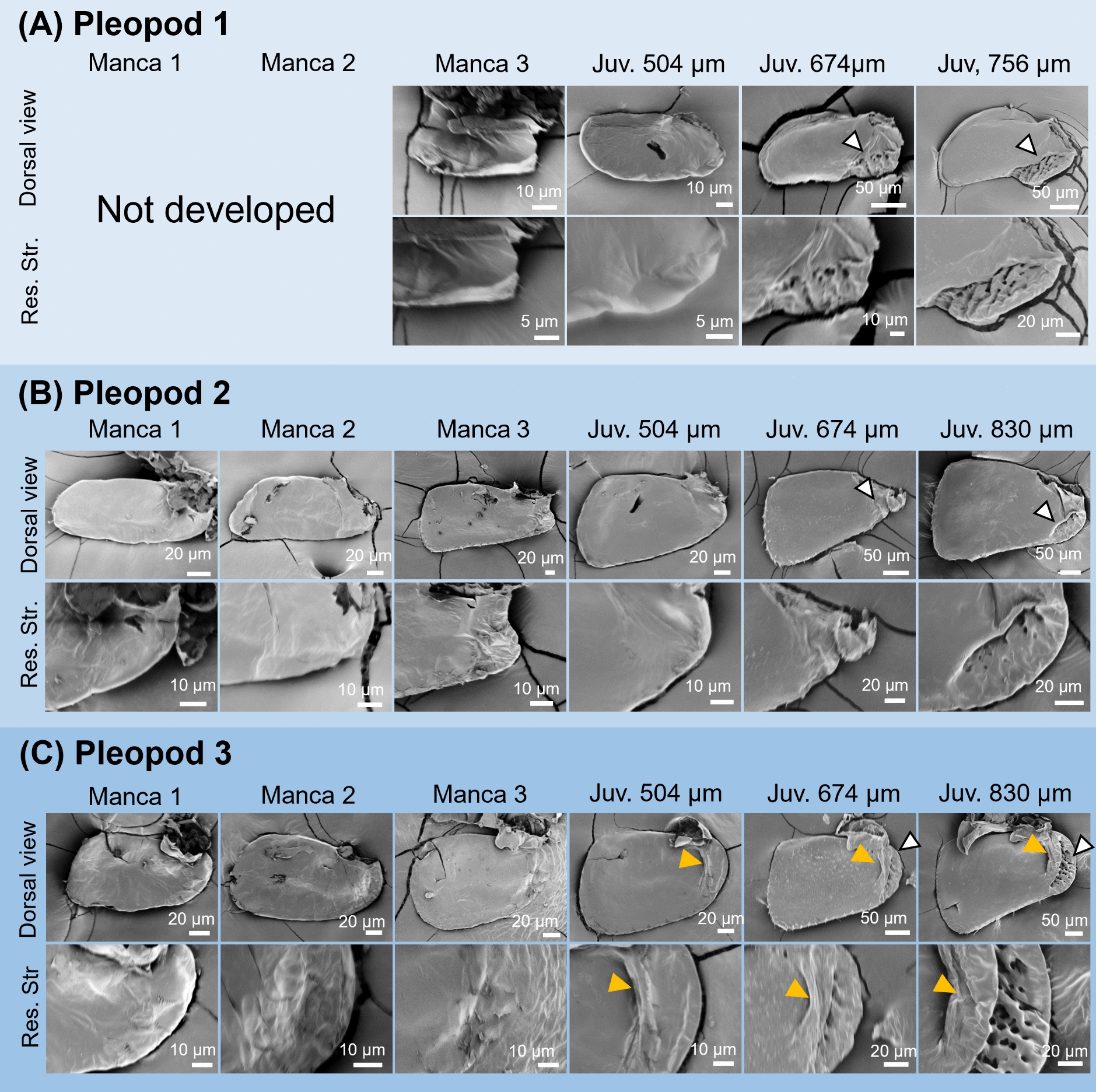
Morphological observations of the first (**A**), second (**B**) and third (**C**) pleopods in *N. okinawaensis* during development. Lengths indicate head width of post-manca juveniles. Arrowheads indicate respiratory structures (white) and swollen ridges (orange). *Juv* post-manca juvenile, *Res* *Str* respiratory structure

SEM observations on the dorsal surface of the pleopods showed that no respiratory structures were present in the second and third pleopods during the manca stages (Fig. 3). Also, no structures were observed in the first pleopod at manca 3 stage, when the first pleopod appeared (Fig. 3A). In the post-manca juvenile stage, a swollen ridge was formed on the proximal lateral side only in the third pleopod (Fig. 3, yellow arrowheads). After these stages, wrinkled surface structures appeared on the proximal side of all pleopods, followed by the appearance of respiratory structures with surface grooves (Fig. 3, white arrowheads). At the stages when they appeared, these structures consisted of a few grooves (Fig. 3). In later stages, the number of grooves increased and the structures became more complex (Fig. 3).

**Fig. 4:**
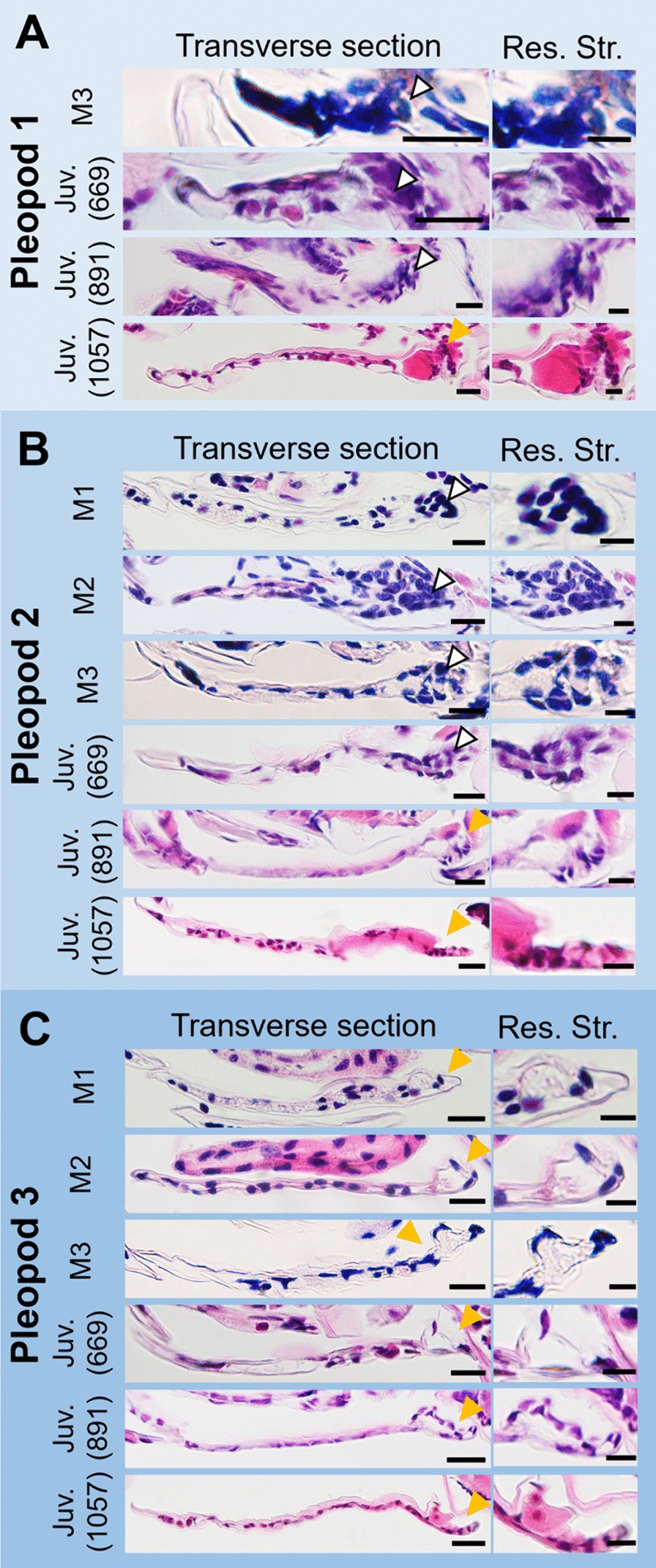
Histological observations of the first (**A**), second (**B**) and third (**C**) pleopods in *N. okinawaensis* during development*.* Arrowheads indicate aggregated cells (white) and proximal hemolymph sinus (orange). Lengths indicate head width of post-manca juveniles. Scale bars indicate 20 μm (Transverse section) and 10 µm (Res. Str.). *Juv* post-manca juvenile, *Res*. *Str* respiratory structure

In transverse sections, a cell-dense region was observed at the proximal side of the pleopods in the first and second pleopods during the manca stages (Fig. 4A–B, white arrowheads). Later, in the post-manca juvenile stages, hemolymph sinuses were observed at the same location, around which the epithelium was partially invaginated (Fig. 4A–B, yellow arrowheads). In contrast, in the third pleopod, the hemolymph sinus was already observed in the manca stages. During development, the surrounding epithelium gradually changed to structures with numerous grooves on the dorsal surface (Fig. 4C, yellow arrowheads). The thickness of the cuticle was the same between the dorsal and ventral surfaces during the manca stages. However, during the post-manca juvenile stages, the cuticle was highly thickened on the dorsal surface, except for the respiratory structures (Fig. 4).

### Dorsal respiratory fields in *Alloniscus balssi*

#### Adult structure

**Fig. 5:**
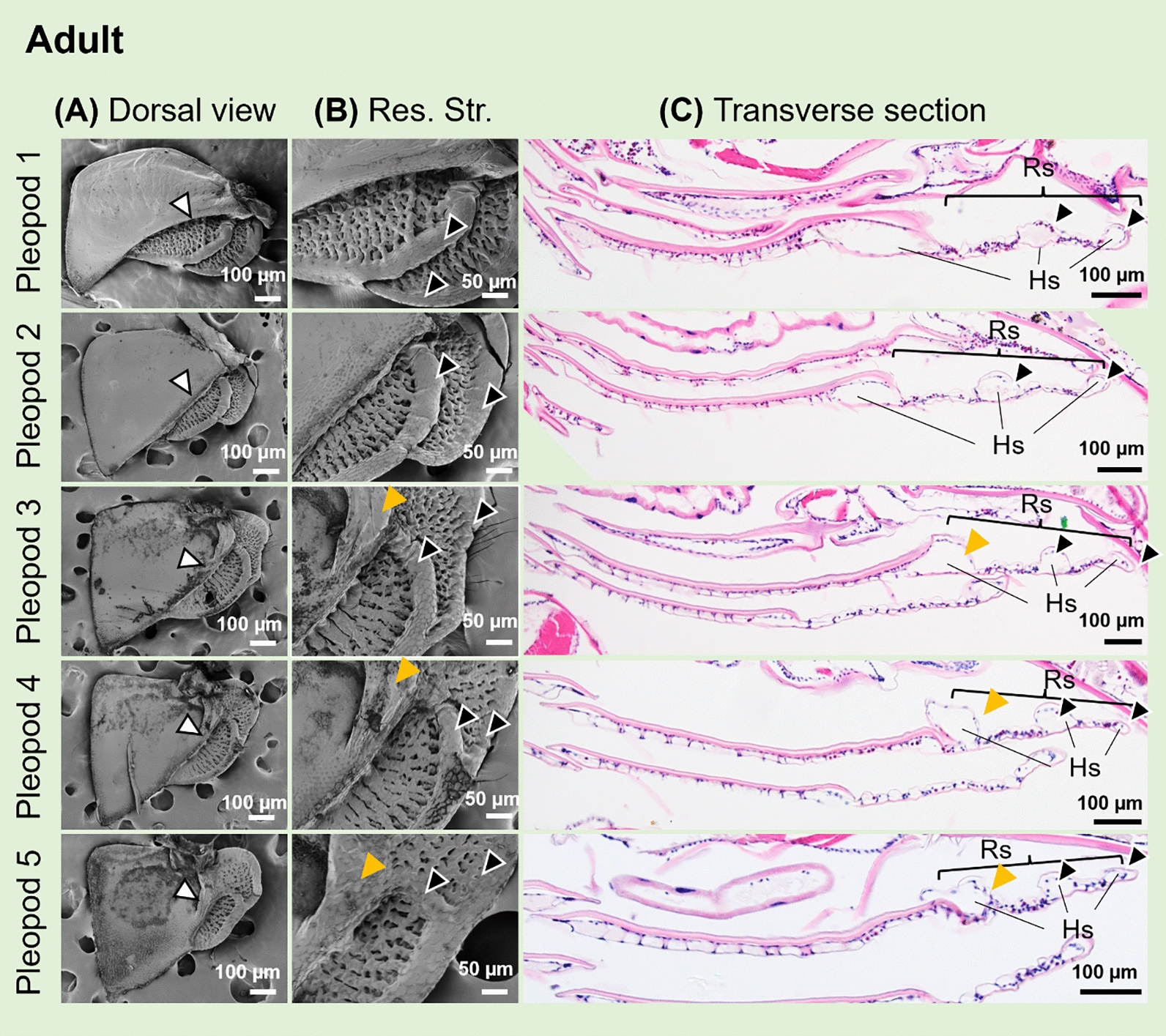
Adult pleopods of *Al. balssi.* **A** Dorsal view of the exopods of the pleopods in SEM images. White arrowheads indicate respiratory structures. **B** Enlarged views of respiratory structures (dorsal respiratory structures). Arrowheads indicate proximal swollen ridges (orange) and other swollen ridges (black). **C** Transverse sections of pleopods. Respiratory structures located in proximal sides. Arrowheads indicate proximal (orange) and other (black) hemolymph sinuses. Crossed arrows indicate dorsal–ventral and proximal–distal axis. *Hs* hemolymph sinus, *Res*. *Str*. and *Rs* respiratory structure

SEM observations on pleopods revealed that adults had slightly wrinkled respiratory structures on the exopods of all pleopods (Fig. 5A, white arrowheads). All structures were located on the proximal lateral side of the pleopods with swollen ridges in the structures (Fig. 5B black arrowheads). In addition, the third to fifth pleopods had distinct swollen ridges at the borders between the structures and the dorsal surface of the pleopods (Fig. 5B, yellow arrowheads).

Observations on transverse sections showed that the dorsal sides of the pleopods were covered by a thick cuticle, while the respiratory structures were covered by a very thin cuticle. Within the structures, three internal hemolymph sinuses were observed (Fig. 5C). All pleopods had similar tissues and the locations of hemolymph sinuses corresponded to the swollen ridges (Fig. 5, yellow and black arrowheads). The ventral side of the pleopods was composed of columnar epithelial cells (Fig. 5C).

#### Developmental processes

The first, second and third pleopods were morphologically observed during the manca stages and the post-manca juvenile stages.

**Fig. 6:**
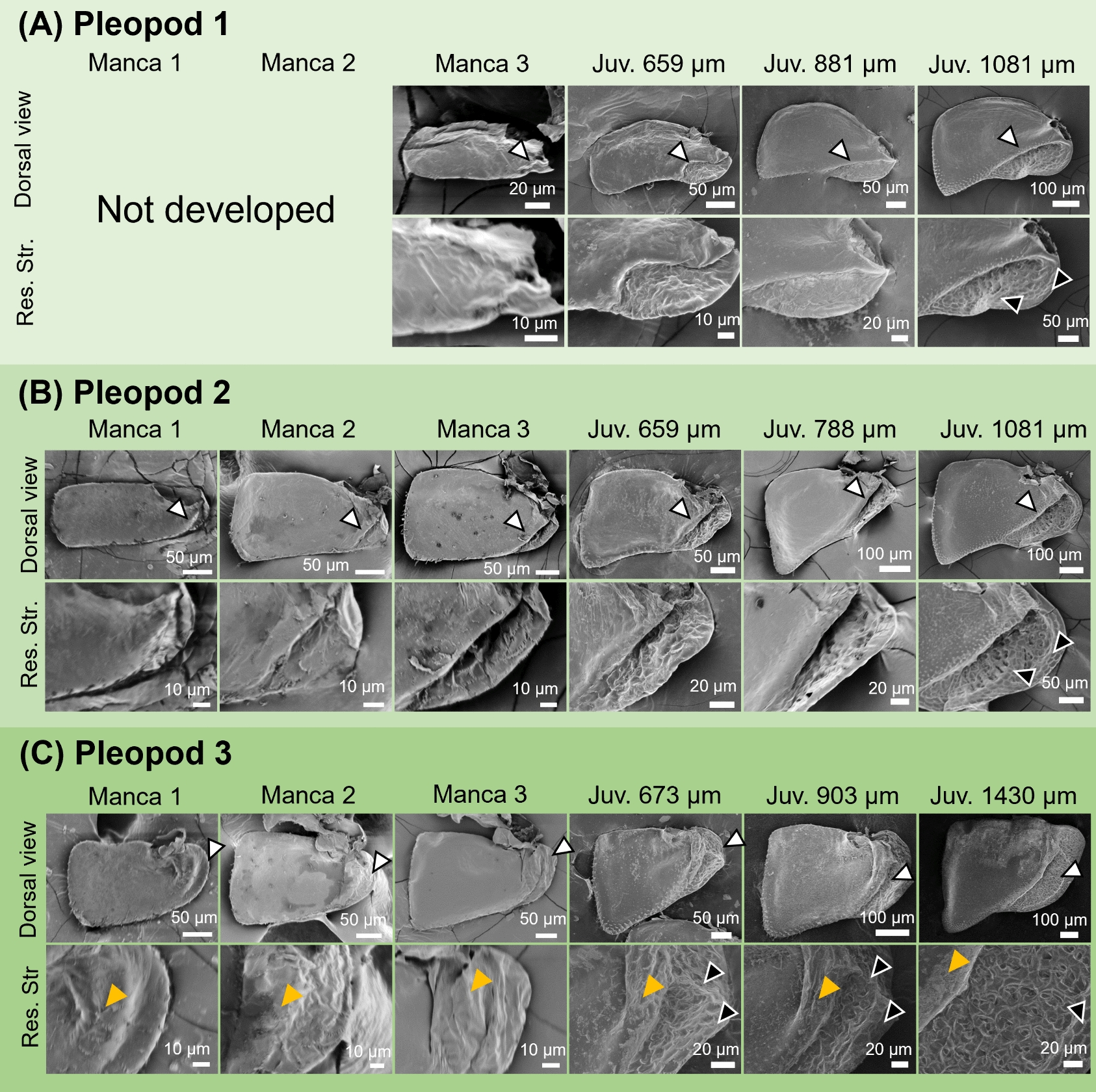
Morphological observations of the first (**A**), second (**B**) and third (**C**) pleopods in *Al. balssi* during development. Lengths indicate head width of post-manca juveniles. Arrowheads indicate respiratory structures (white), proximal swollen ridges (orange) and other swollen ridges (black). *Juv* post-manca juvenile, *Res*. *Str* respiratory structure

SEM observation on the dorsal surfaces of pleopods revealed that primordial regions where the respiratory structure is formed were already present in the manca stages (Fig. 6, white arrowheads; identification of these areas as respiratory structures was also confirmed by the following histological observations.). These regions were separated from the dorsal surface by the lateral sides of the pleopods and became enlarged and wrinkled during development (Fig. 6). As the regions expanded, swollen ridges developed on the lateral side and in the center of the regions (Fig. 6, black arrowheads). In addition, another swollen ridge was observed near the proximal side of the third pleopod in the manca stages (Fig. 6C, yellow arrowheads).

**Fig. 7:**
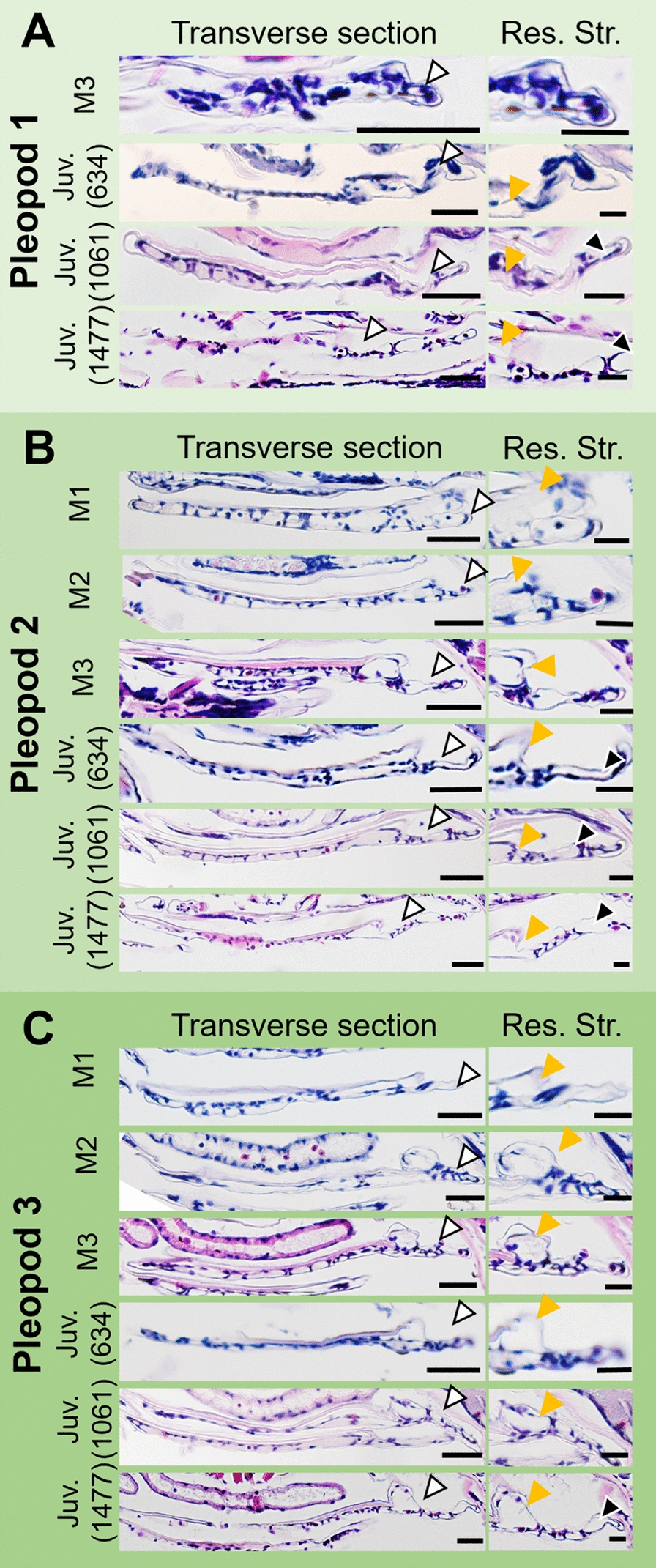
Histological observations of the first (**A**), second (**B**) and third (**C**) pleopods in *Al. balssi* during development*.* Arrowheads indicate laterally protruding epithelial tissue (white), proximal hemolymph sinus (orange) and other hemolymph sinuses (black). Lengths indicate head width of post-manca juveniles. Scale bars indicate 50 μm (Transverse section) and 20 µm (Res. Str.). *Juv* post-manca juvenile, *Res*. *Str* respiratory structure

Histological observations showed that the ventral epithelium of the pleopods protruded laterally in the manca stages (Fig. 7, white arrowheads). The protrusions were composed mainly of columnar epithelial cells and corresponded to the primordial respiratory region on the surface (Fig. 6). These protruding epithelial tissues expanded with growth (Fig. 7). In the first pleopods, proximal hemolymph sinuses were observed during the post-manca juvenile stages (Fig. 7A, yellow arrowheads). In the second and third pleopods, the proximal hemolymph sinuses were observed during the manca stages (Fig. 7B–C, yellow arrowheads), and the epithelium around these hemolymph sinuses did not cause major structural changes. As the protruding ventral epithelium expanded and increased in size, additional hemolymph sinuses were observed in the structures (Fig. 7, black arrowheads).

### Pleopods of *Armadillidium* cf.* ellipticus*

**Fig. 8:**
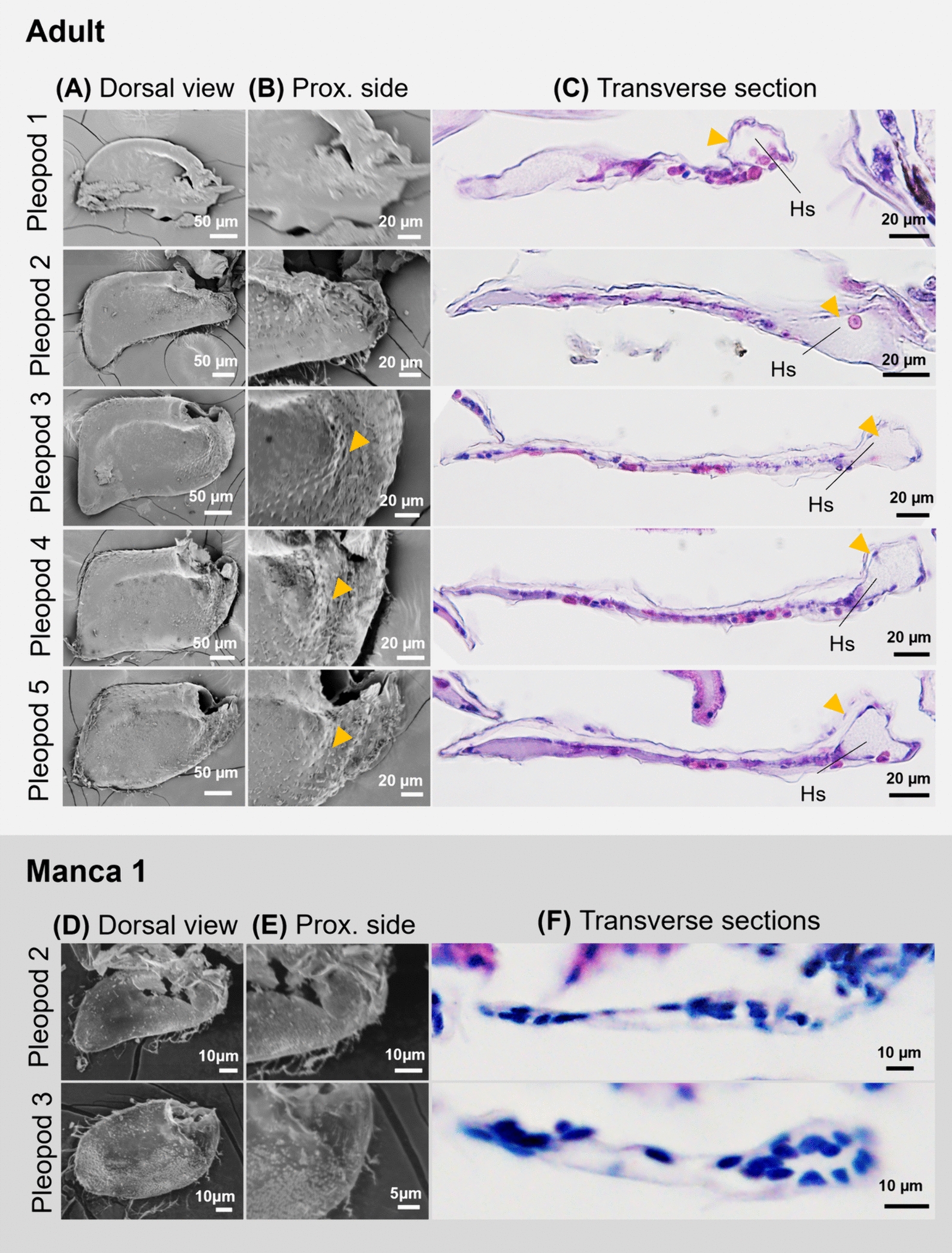
Adult and manca 1 pleopods of *Ar.* cf. *ellipticus.* **A, D** SEM observation. Dorsal view of the exopods of the pleopods in adults (**A**) and manca 1 (**D**). **B, E** Enlarged views of the proximal side in adults (**B**) and manca 1 (**E**). Arrowheads indicate swollen ridges. **C, F** Transverse sections of the pleopods in adults (**C**) and manca 1 (**F**). Arrowheads indicate proximal hemolymph sinus. Crossed arrows indicate dorsal–ventral and proximal–distal axis. *Hs* hemolymph sinus

Finally, to compare the previous results with the structures in a species lacking special respiratory structures, the pleopods of *Ar.* cf. *ellipticus* were examined in an adult and at manca 1 stage (Fig. 8). Although no respiratory structures developed on the proximal lateral side of the pleopods, fine scale-like surface structures were observed on the third, fourth and fifth pleopods (Fig. 8B). In addition, swollen ridges were observed on the third to fifth pleopods (Fig. 8B, yellow arrowhead). Observations of histological sections revealed that hemolymph sinuses were located in the proximal sides of all pleopods (Fig. 8C, yellow arrowhead). The cuticle was thicker in the dorsal epithelium of all pleopods (Fig. 8C).

At manca 1 stage, no obvious surface structures were observed (Fig. 8D-E), but a cell-dense regions was observed (Fig. 8F) where hemolymph sinuses occur in adults (Fig. 8C). No differences in thickness between dorsal and ventral cuticles were observed at this stage.

## Discussion

### Comparison of developmental processes between uncovered lungs and dorsal respiratory structures

In previous studies, adult morphologies have been described mainly in species of the genus *Trachelipus* (Trachelipodidae) for uncovered lungs [15, 21] and in the genus *Oniscus* (Oniscidae) for dorsal respiratory fields [14, 15, 22]. In this study, the detailed developmental processes were described focusing on uncovered lungs in *N. okinawaensis* and on dorsal respiratory fields in *Al. balssi*, in comparison with pleopod structures of the basal lineage *Ar.* cf. *ellipticus*.

**Fig. 9:**
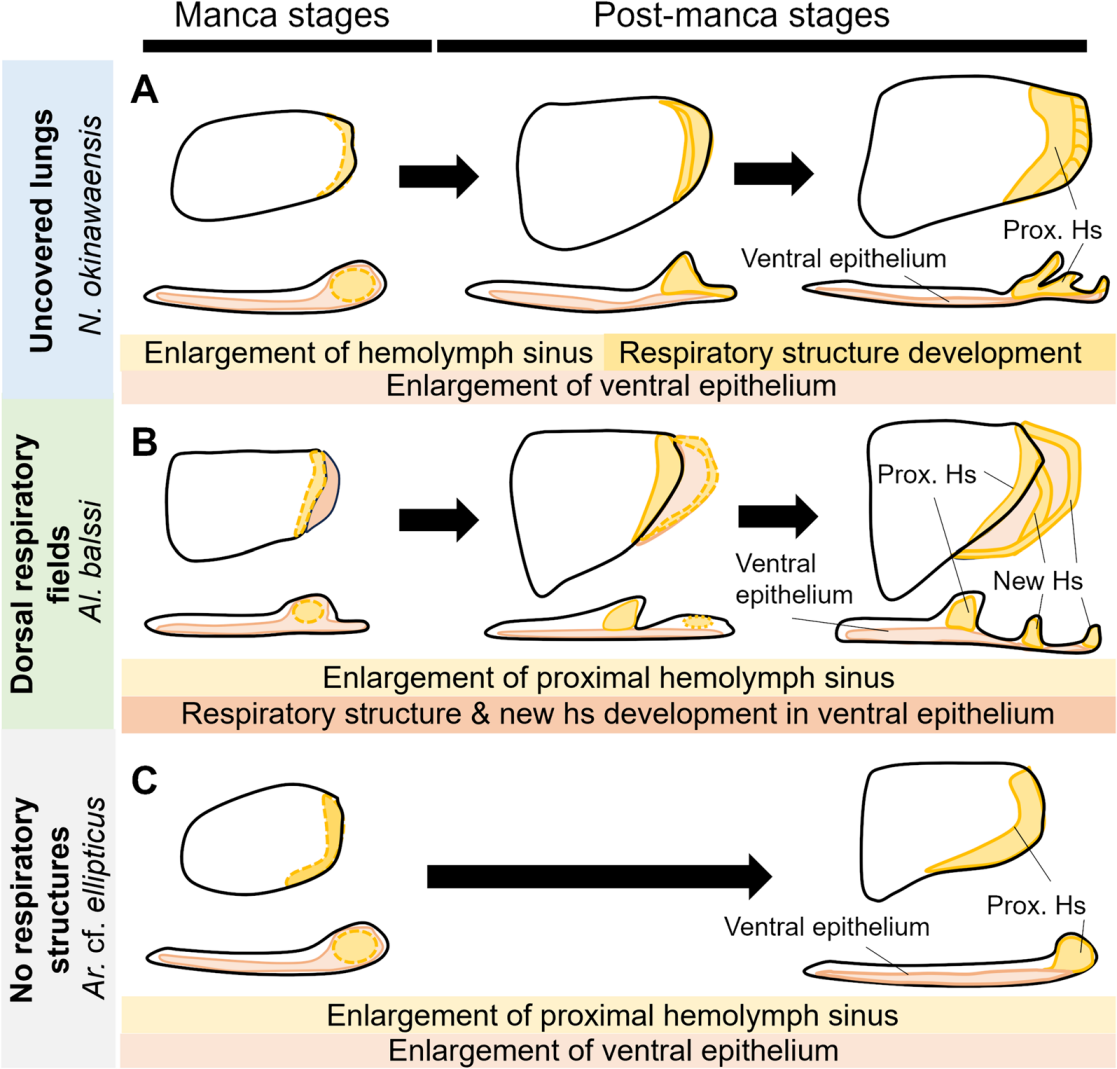
Summary of respiratory structure development in focal isopod species. The exopods of the third pleopod are illustrated as representatives. Dorsal views and transverse sections are shown for each stage. **A** Uncovered lungs development in *N. okinawaensis*. **B** Dorsal respiratory fields development in *Al. balssi*. **C** Pleopodal development in *Ar.* cf. *ellipticus*. *Hs* hemolymph sinus, *Prox* proximal

In *N. okinawaensis*, the location of the respiratory structures and the hemolymph sinuses also corresponded to those of *Trachelipus* [14, 15]. Although the proportion of covered area was smaller than in *Trachelipus* [14, 15], the adult respiratory structures in *N. okinawaensis* were generally consistent with the features of ‘uncovered lungs’ in *Trachelipus* species. Uncovered lungs of *N. okinawaensis* developed mainly by modification of the lateral epithelium around the proximal hemolymph sinus (Fig. 9A). Histological observations suggested that the wrinkled respiratory surfaces and tubular structures in the groove are formed by epithelial invagination (Fig. 3).

The adult respiratory structures in *Al. balssi* revealed in this study agreed with the previous descriptions of dorsal respiratory fields in the genus *Oniscus*, except for the number of hemolymph sinuses [14, 15, 22]. In this species, the regions that would become the respiratory structure were observed at manca 1 stage, immediately after hatching. These regions were formed by the lateral protrusion of the ventral epithelium, which gradually became surface wrinkles (Fig. 9B).

Furthermore, observations in *Ar.* cf. *ellipticus* suggested that in the species lacking respiratory structures, only the proximal hemolymph sinuses and the dorsal cuticle developed during postembryonic development (Fig. 8; Fig. 9C).

The formation of a proximal hemolymph sinus and a thickened dorsal cuticle were shared processes in all species (Fig. 3; Fig. 6; Fig. 8). In addition, swollen ridges developed on the third through fifth pleopods with the formation of the hemolymph sinus. The previous studies suggested that these swollen structures may play a role in maintaining moisture and fluids around the endopods [15, 17].

For uncovered lungs in *Nagurus*, this study showed that the lateral margin/semilunar area, a region homologous to the respiratory structure proposed in previous studies [14, 15, 17], is transformed into the respiratory structure during its development. On the other hand, for the dorsal respiratory fields in *Alloniscus*, the developmental origin of the respiratory structure is not the lateral epithelium of the hemolymph sinus, but a laterally protruding ventral epithelium (Fig. 9B-C). It is known that the hemolymph flow around the respiratory structures largely differs between dorsal respiratory fields in *Oniscus* and uncovered lungs in *Trachelipus* [18]. Although the species examined were different, these differences were also supported by the observations in the present study.

### Evolutionary origins of respiratory structures in terrestrial isopods

Among Crinocheta lineages with different types of air-breathing organs, the Olibrinidae or Detonidae are proposed to be the most basal clades, and the Trachelipodidae and Porcellionidae are derived clades [14, 23; Fig. 1]. Although traditional phylogenies are based on morphological characters [23], the monophyly of Crinocheta and the phylogenetic relationships are supported to some extent by a molecular phylogenetic analysis [24, 25].

In terrestrial isopods, gas exchange occurs mainly in the exopods of pleopods [13]. In Crinocheta, basal species inhabiting wet coastal and subterranean environments do not have specialized respiratory structures [13]. In these species, gas exchange is suggested to occur through the thin ventral epithelium of exopods [14, 22].

**Fig. 10:**
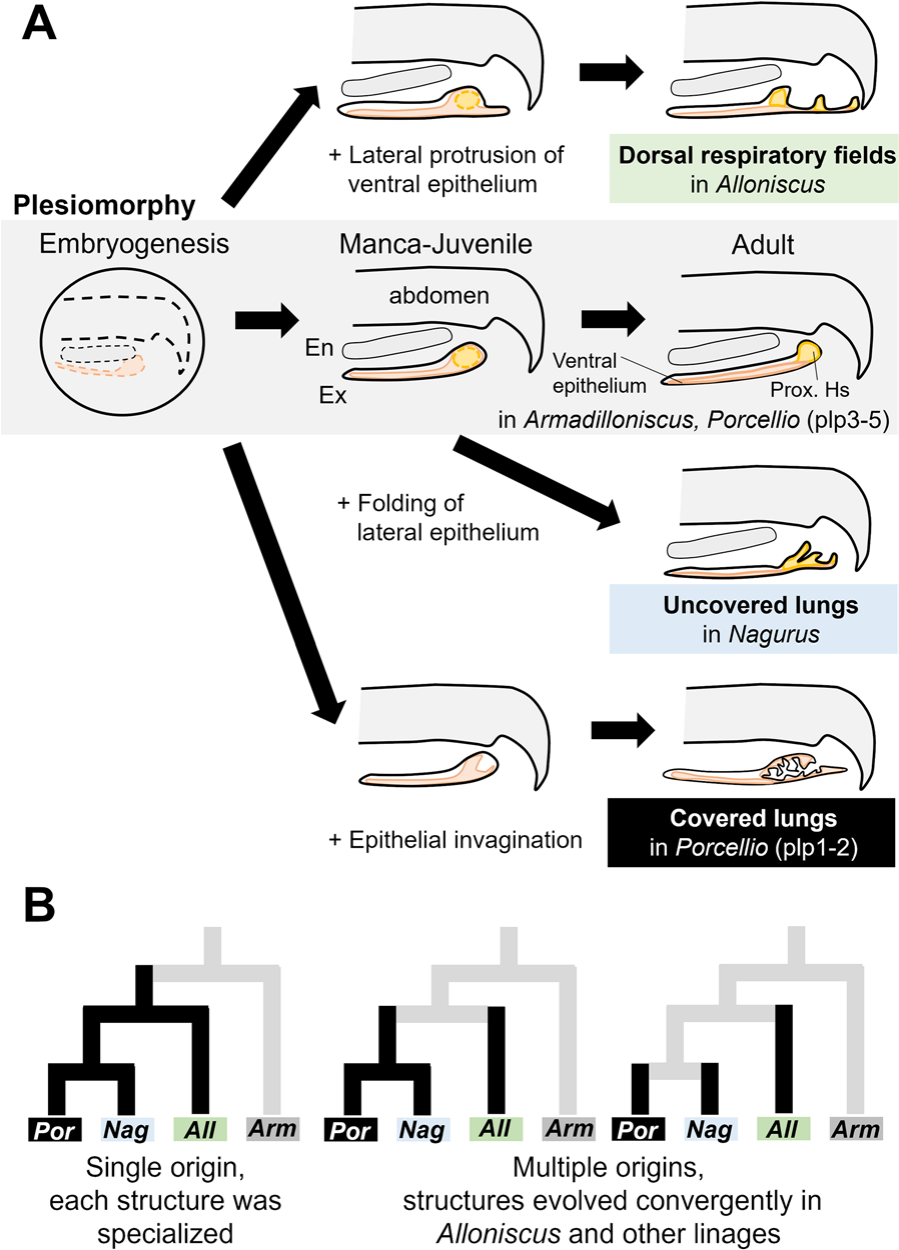
Evolution of novel respiratory structures in isopods. **A** Developmental modification in each lineage. Pleopods are illustrated as transverse view. Arrows indicate direction of development. Structures and developmental processes of covered lungs in *Porcellio scaber* are based on previous studies [14, 20]. **B** Possible hypotheses about the evolutionary origins of isopod respiratory structures. Phylogenetic relationships were based on previous studies [23, 25]. *All* *Alloniscus*, *Arm* *Armadirroniscus*, *Ex* exopod, *En* endopod, *Nag* *Nagurus*, *plp* pleopods, *Por* *Porcellio*

Considering these findings, the observations in *Armadilloniscus* in this study allow us to hypothesize that in the ancestral developmental process of the exopods, only the development of the hemolymph sinus and thickening of the dorsal cuticle occurred during postembryonic development (Fig. 10A). Thus, the respiratory structure of terrestrial isopods was acquired by the addition of novel developmental processes during evolution.

For dorsal respiratory fields, the tissue structure of the pleopods differs from the assumed ancestral pleopod at the manca 1 stage, suggesting that specific developmental changes occurred at manca 1 stage or late in embryonic development. The thin ventral epithelium, which was responsible for gas exchange in the ancestor, protrudes laterally to expand the respiratory surface (Fig. 10A). On the other hand, in the uncovered lung, it is assumed that the ancestral developmental process is preserved until a certain point in the post-manca stage, but then a new developmental process is acquired that produces folding and depression of the lateral epithelium around the proximal hemolymph sinus (Fig. 10A). Although it is unclear whether this lateral epithelium originally contributed to gas exchange, the increased size of the epithelium around the sinus probably contributes to the efficiency of respiration.

Furthermore, considering the developmental processes in covered lungs of *Porcellio scaber* [20], it is thought that at least one specific change in the developmental mechanism occurs at early manca 1 stage in covered lungs (Fig. 10A). It is difficult to specify the position of the epithelium that becomes the lungs because the epithelial invagination occurs during a single developmental stage and the complete lung is established at the next manca stage [20].

These changes of postembryonic development in pleopods would be related to efficient gas exchange under the conditions of a fully developed exoskeleton with the cuticle. It has been suggested that the lungs of terrestrial isopods may have evolved with the impermeability of the exoskeleton for water retention [26, 27], and a similar phenomenon would occur during their ontogeny.

It is known that some evolutionary novelties can be acquired by the modification of developmental mechanisms already present in the ancestor [28]. Therefore, there are still multiple evolutionary scenarios on the acquisition of these respiratory structures that could have derived from a single origin or from multiple origins (Fig. 10B). The differences in epithelial positions developing into respiratory structures between the dorsal respiratory fields of *Alloniscus* and the uncovered lungs of *Nagurus* suggest the possibility that the heterotopic changes of developmental mechanism or independent acquisition. Covered lungs in *Porcellio* species and uncovered lungs in *Nagurus* differ in the number of the respiratory structures, as well as the timing and mode of development. However, given that the Trachelipodidae and the Porcellionidae are closely related [24, 25] and have similar epithelial modifications, the possibility that covered lungs and uncovered lungs share the origin could not be excluded (Fig. 10B). To get insights into the homology more accurately, detailed investigations like  cell or tissue tracking using molecular markers should be required in future studies.

In terrestrial amphipods, it has been shown that molecular developmental changes contributed to the thickening of their gills during this expansion into montane environments [29]. Although amphipods did not evolve lungs, terrestrial isopods should also have some novel molecular developmental mechanisms related to the acquisition of respiratory structures. Analysis of molecular mechanisms and observations of more lineages are needed to elucidate the detailed evolutionary processes of isopod respiratory structures.

## Conclusions

This study shows that the respiratory structures in terrestrial isopods develop mainly during postembryonic development, but the timing and mode of development largely differ among the types of respiratory organs. In particular, the developmental origins of the respiratory structures were different between the two species *Nagurus* and *Alloniscus*: in the former, uncovered lungs originated from the epithelium of the lateral surface around the proximal hemolymph sinus of the pleopod, while in the latter, dorsal respiratory fields originated from the ventral epithelium of the pleopod. This would suggest that morphologically different types of isopod respiratory structures do not result from the simple degree of development. To understand the evolutionary processes of these structures, it is necessary to consider heterotopic modifications of developmental mechanisms or different evolutionary origins. Overall, this study provides the basis for evolutionary developmental studies of air-breathing organs in terrestrial isopods and insights into their evolutionary divergence and convergence.

## Materials and methods

### Animals

Samples of *N. okinawaensis* were collected from the soil or plantings on the streets of Naha city and around the University of Ryukyu campus, on Okinawa Island, Japan in November 2022. Samples of *Al. balssi* and *Ar.* cf. *ellipticus* were collected from the seashore around the Misaki Marine Biological Station campus in 2021–2023. Species were identified based on previous studies [30–33]. Although the *Armadilloniscus* species at the site where we collected *Ar.* cf. *ellipticus* were previously identified as *Ar. japonicus* Nunomura, 1984 [32], it was subsequently suggested that this species was probably the same as *Ar. ellipticus* [34]. Therefore, this species was treated as *Ar.* cf. *ellipticus* in the present study. Some of the collected isopods were kept in plastic cases filled with moistened fallen leaves to obtain ovigerous females according to a previous study [20]. Mancae, post-manca juveniles, and adults were observed under a stereomicroscope (SZX16; Olympus, Tokyo, Japan) equipped with a digital camera (DP50; Olympus, Tokyo, Japan) to distinguish their developmental stages. Stages of mancae were defined following a previous study [20]. Their head width was measured as an indicator of approximate body size according to a previous study [35] because it is difficult to define the stages of post-manca juveniles based on morphology. For post-manca juveniles and adults, only females were used for observation due to their sexual dimorphisms in pleopods [36].

### Scanning electron microscopy

Scanning electron microscopy (SEM) was employed to observe the external morphology of the respiratory structures during development. The samples were prepared according to the methods described in a previous study [20]. The exopods of the first, second, and third pleopods were observed under a scanning electron microscope (JSM-5510LV; JEOL Ltd., Tokyo, Japan).

### Histological observations

To observe the development processes of the respiratory structures histologically, paraffin sections were made basically according to a previously described method [20]. The whole bodies were fixed in Bouin’s solution for several days and preserved in 70% EtOH. The samples were dehydrated by immersion in increasing concentrations of ethanol, transferred to xylene, and finally embedded in paraffin. Serial sections of the transverse planes were prepared using a microtome (Spencer Lens Co., Buffalo, USA). The thickness of section slices was 8 μm. After deparaffinization, the sections were stained with hematoxylin and eosin. The tissues on the slides were observed under a microscope (BX51; Olympus, Tokyo, Japan) equipped with a digital camera (DP74; Olympus, Tokyo, Japan). All of the continuous sections were observed in detail, and the images with the maximum degree of respiratory structures are shown in figures.

## Data Availability

All data used and analyzed during the study are available from the corresponding author on reasonable request.
